# The Duration of Gastrointestinal and Joint Symptoms after a Large Waterborne Outbreak of Gastroenteritis in Finland in 2007 – A Questionnaire-Based 15-Month Follow-Up Study

**DOI:** 10.1371/journal.pone.0085457

**Published:** 2014-01-17

**Authors:** Janne Laine, Jukka Lumio, Salla Toikkanen, Mikko J. Virtanen, Terhi Uotila, Markku Korpela, Eila Kujansuu, Markku Kuusi

**Affiliations:** 1 Tampere University Hospital, Department of Internal Medicine, Tampere, Finland; 2 National Institute of Health and Welfare, Epidemiologic Surveillance and Response Unit, Helsinki, Finland; 3 University of Tampere, School of Medicine, Tampere, Finland; 4 Nokia Health Centre, Nokia, Finland; Kliniken der Stadt Köln gGmbH, Germany

## Abstract

An extensive drinking water-associated gastroenteritis outbreak took place in the town of Nokia in Southern Finland in 2007. 53% of the exposed came down with gastroenteritis and 7% had arthritis-like symptoms (joint swelling, redness, warmth or pain in movement) according to a population-based questionnaire study at 8 weeks after the incident. *Campylobacter* and norovirus were the main pathogens.

A follow-up questionnaire study was carried out 15 months after the outbreak to evaluate the duration of gastrointestinal and joint symptoms. 323 residents of the original contaminated area were included. The response rate was 53%. Participants were inquired about having gastroenteritis during the outbreak and the duration of symptoms.

Of those with gastroenteritis, 43% reported loose stools and abdominal pain or distension after the acute disease. The prevalence of symptoms declined promptly during the first 3 months but at 15 months, 11% reported continuing symptoms. 32% of the respondents with gastroenteritis reported subsequent arthritis-like symptoms. The disappearance of arthritis-like symptoms was more gradual and they levelled off only after 5 months. 19% showed symptoms at 15 months. Prolonged gastrointestinal symptoms correlated to prolonged arthritis-like symptoms.

High proportion of respondents continued to have arthritis-like symptoms at 15 months after the epidemic. The gastrointestinal symptoms, instead, had declined to a low level.

## Introduction

Although acute gastroenteritis is a common disease, little is known about its consequences after passing the acute phase of the disease. As nearly everyone meets episodes of acute gastroenteritis occasionally, the possible connection with later health problems is easily missed. Food- and waterborne epidemics give special opportunity to study this connection prospectively as a cohort of people is infected in a relatively short period, often with identified pathogens. Epidemics large enough to conduct a study are, however, uncommon in developed countries with a good level of investigation capabilities.

Reactive arthritis (ReA) and milder forms of joint complaints are well known and fairly common acute complications of bacterial gastroenteritis [1]. There is also growing evidence of increased incidence of irritable bowel syndrome (IBS) after bacterial [2], [3] and possibly viral [4] gastroenteritis. The most intensively studied waterborne outbreak is the Walkerton epidemic in Ontario, Canada. According to the Walkerton Health Study, increased risk of reactive arthritis, irritable bowel syndrome, pregnancy-related hypertension, hypertension, kidney disease, and even cardiovascular events has been observed after the epidemic [5], [6], [7], [8], [9], [10].

A large drinking-waterborne epidemic occurred in a Finnish town Nokia in November–December 2007. Shortly after the incident, a comprehensive outbreak investigation of short- and long-term health effects was initiated. Details on the epidemiology, microbial findings, and early arthritic complaints have been published previously [11], [12], [13], [14], [15]. We performed a questionnaire study at 15 months after the exposure to contaminated water to observe the duration of symptoms and the remaining health complaints at 15 months after the incident. Here, we present data on the persistence of gastrointestinal and joint symptoms after the outbreak.

## Methods

### Setting

The town of Nokia is located in Southern Finland and has a population of 30 000. At the end of November 2007, maintenance work was carried out in the town's wastewater plant. During the work, a valve connecting the wastewater plant's effluent line and household water distribution line was opened and it was accidentally left open for two days. Approximately 450 m^3^ of plant's effluent water contaminated the drinking water of 9 500 residents. This resulted in a large gastroenteritis outbreak in the contaminated area of the town. Some excess morbidity was also detected in the uncontaminated area [11]. The epidemic peaked four days after the incident, and most gastroenteritis cases occurred within two weeks.

Seven pathogens were found from the patients' stools. Six of those were also detected from tap water or water-distribution network samples. *Campylobacter* was the most common pathogen found in 27% (N = 148) of stool samples. In addition, norovirus was also considered as a major pathogen [16]. Non-typhoidal *Salmonellae* and *Giardiae* [13] were detected in less number of samples. In a study focusing on children with gastroenteritis, other viruses and infections with mixed pathogens were also observed [12].

According to a population-based questionnaire survey performed eight weeks after the incident 53% of the population in the contaminated area reported falling ill with gastroenteritis and 6.7% reported having arthritis-like joint symptoms [15]. In spite of active encouragement to refer new cases of probable arthritis to a rheumatologist at the local university hospital, only 21 confirmed cases of ReA were detected [14].

### Questionnaire studies

Two consecutive questionnaires were mailed 13 months apart. The first was mailed eight weeks after the incident to detect the immediate morbidity [15]. The second, a follow-up study, was performed fifteen months after the outbreak in order to study the persistence of gastrointestinal and joint symptoms.

Details of the first questionnaire at 8 weeks were presented in previous articles [11], [15]. In brief, two target populations were defined from Nokia; those residing in the part of town with contaminated water supply (“contaminated group”) and those residing in the uncontaminated area (“uncontaminated group”). A third group (“control group”) was chosen among the citizens of another municipality in the same district. Approximately, 1000 participants were randomly picked from the national population registry for each group (Table 1). All the ages were included and the groups were matched for age and gender. Only one participant per household was allowed.

**Table 1:** Evolution of the study groups in the follow-up study.

	Nokia,contaminated	Nokia,uncontaminated	Control population
Population 2007	9 538	20 478	27 259
Size of the study groups in the first study	1 021	979	1 000
Responded to the first study	808	717	598
Sample size in the follow-up study (i.e., those who gave permission to be contacted again)	615	498	343
Responded to the follow-up study	323	230	186
Gastroenteritis during the epidemic, according to the follow-up study	174		

Study groups were based on the original population samples that were used in the first survey. The study groups became step-wise smaller because of lack of response to the first survey, denying further contact, and lack of response to the follow-up survey.

The second questionnaire at 15 months (“follow-up”) was mailed to persons who had participated in the first survey and given their consent for being contacted again. Therefore, the groups became smaller (Table 1.). No reminder was mailed to those who did not respond. Participants were asked whether they had gastroenteritis during the outbreak period (from November 28 to December 31 2007). They were also asked for how long (weeks or months) abdominal or joint symptoms persisted after the acute gastroenteritis.

Gastrointestinal symptoms, such as the presence of loose stools, constipation, nausea, abdominal pain, and abdominal distension were recorded. After analyzing these symptoms separately, a combination of gastrointestinal symptoms was created to observe IBS-like symptoms, such as loose stools and abdominal pain or distension. This combination is close to the Manning 3 and/or Rome I IBS-criteria [17].

Of joint symptoms, joint pain, pain in joint movement and the presence of swelling, redness, or warmth of the joint were recorded. The condition was classified as arthritis-like if pain in joint movement or any of the symptoms of joint swelling, redness, or warmth was present as defined in our previous study of early joint symptoms [15].

The persistence of gastrointestinal and arthritis-like symptoms was then analyzed as the remaining prevalence of a symptom in the course of time among those who had declared as having had gastroenteritis during the epidemic.

### Use of microbiological data

Data of the stool specimens sent for microbiological analysis were obtained from the database of the Fimlab laboratories. This institution is a district-wide and public-run clinical laboratory serving the entire public health-care sector in the district. From year 2005 to 2009, the quantity, dates, and microbiological findings among the residents of Nokia and the control municipality were collected for comparison.

### Statistical methods

Completed forms were stored in a database and analysed in the National Institute of Health and Welfare. Estimation of the effect of selection was done in two steps. First, the probability to be included in the study (i.e., those who gave permission to be contacted again) within all the initial study subjects was estimated by using univariate logistic regression. Secondly, the probability of returning the questionnaire within the included subjects was estimated in a similar manner. Confidence intervals for the proportion of subjects continuing to have symptoms at each time point were calculated by using ordinary bootstrap. All analyses were done with R version 2.15.2.

### Ethical considerations

When answering the first questionnaire, participants were asked whether they would accept a follow-up contact. An informed consent was attached in the second questionnaire. The follow-up study was approved by the ethical committee of the Tampere University Hospital.

## Results

### Response rates and selection

Answer on the first questionnaire was received from 808 (79%) persons of the contaminated group, 717 (73%) of the uncontaminated group and 598 (60%) of the control group (Table 1). Taking all groups together, 667 (31.4%) of those responding declined the request for further contact. Response rates to the follow-up questionnaire were 53% (323/615), 46% (230/498), and 54% (186/343), respectively.

The odds ratios (ORs) for probability to be included in the follow-up questionnaire (i.e., giving permission to be contacted again) were 1.2 (95% CI 1.0–1.5) for the female gender, 1.7 (1.3–2.1) for those who had reported gastroenteritis and 1.7 (1.3–2.2) for those reporting joint symptoms in the first questionnaire. For these features the ORs for responding to the follow-up questionnaire were 1.1 (0.9–1.3), 1.0 (0.8–1.2) and 1.03 (0.8–1.3), respectively.

Of those participating in the follow-up study, 54% (174/323) in the contaminated group, 13% (31/230) in the uncontaminated group, and 3% (6/186) in the control group reported that they had gastroenteritis during the outbreak.

### Gastrointestinal symptoms

**Figure 1:**
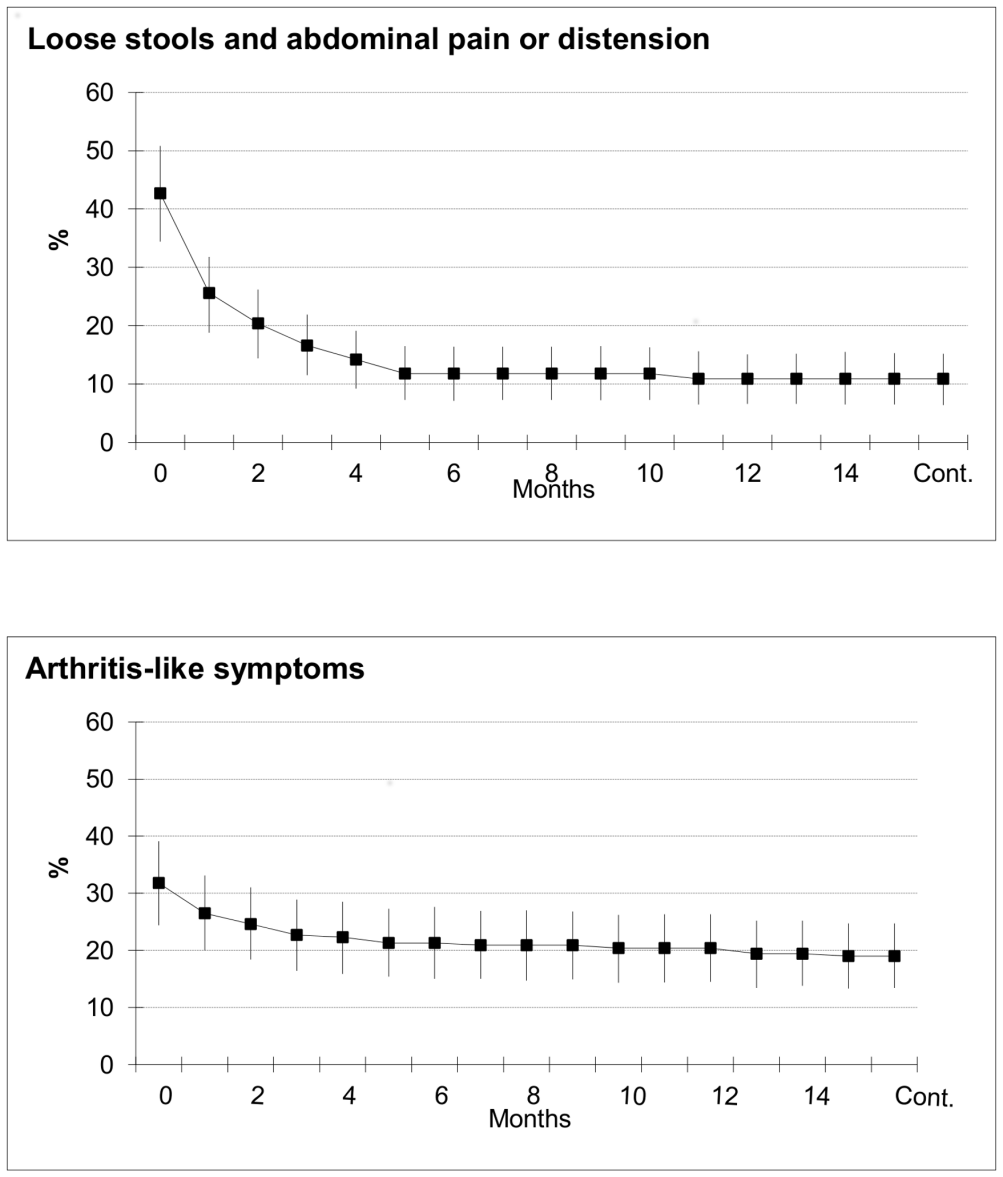
The prevalence of gastrointestinal and arthritis-like symptoms in the contaminated group within fifteen months from the outbreak. The proportions were counted as dividing the number of subjects with symptoms by the number of subjects having fallen ill with gastroenteritis at the time of the outbreak (N = 174). The curves represent the proportion of subjects with symptoms and the vertical lines are the 95% confident intervals.

Of the 174 persons in the contaminated group who had gastroenteritis, 53.6% (93) reported continuing loose stools, 47.9% (83) abdominal pain, and 32.7% (57) abdominal distension after the acute disease. 42.7% (74) experienced loose stools and abdominal pain or distension in the beginning of the follow-up (Figure 1). After a rapid decrease in the proportion of symptomatic persons in one month, the decline started to level off. At 15 months, 10.9% (19) of the respondents continued to have loose stools and abdominal pain or distension. This figure was 11.5% for adults 16 years of age or older and 8.9% for children.

### Arthritis-like symptoms

Among the 174 participants in the contaminated group who had experienced gastroenteritis, 31.8% (55/174) reported arthritis-like symptoms after acute gastroenteritis. The proportion of persons with arthritis-like symptoms declined more slowly over time than the corresponding proportion with gastrointestinal symptoms (Figure 1). At 15 months, 19.0% (33/174) reported as continuing to have arthritis-like symptoms. The persistence of gastrointestinal symptoms (loose stools and abdominal pain or distension) predicted the presence of arthritis-like symptoms at 3 and 15 months (Table 2).

**Table 2:** The prevalence of arthritis-like symptoms among participants with and without gastrointestinal symptoms (loose stools and abdominal pain or distension) at 3 and 15 months after the water contamination.

	At 3 months	At 15 months
With gastrointestinal symptoms	54.3% (19/35)	52.2% (12/23)
Without gastrointestinal symptoms	10.9% (6/55)	10.4% (7/67)
*p*-value	<0.0001	<0.0001

### Use of microbiological tests

**Figure 2:**
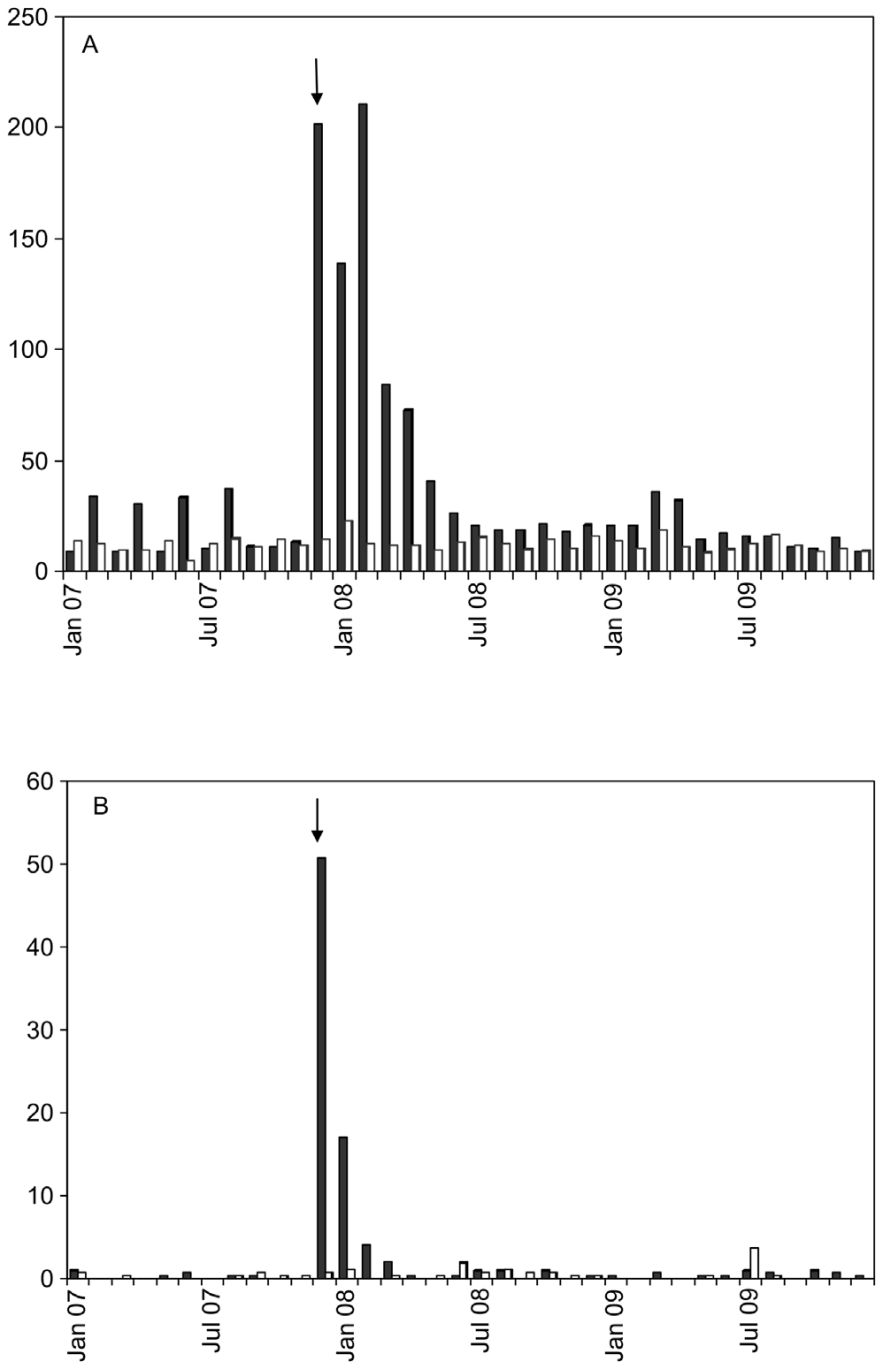
a–b. Monthly incidence (/10 000 inhabitants) of microbiological stool specimens (Fig. 2a) and positive findings (Fig. 2b). Black bars represent the town of Nokia and white bars the control municipality. The arrows indicate the outbreak month, December 2007. Only findings of *Campylobacter* and *Giardia* from January 2007 to December 2009 were counted.

In Nokia, a sharp peak in the number of microbiological stool specimens was observed during the outbreak. Thereafter, the number of specimens diminished gradually and reached the pre-outbreak level in six months. In Nokia, the incidence of positive findings was high during the outbreak and for two months thereafter, but no difference was observed compared to the control municipality after one month (Figure 2).

## Discussion

The faecal contamination of household water and the subsequent large epidemic of gastroenteritis was an exceptional accident [11]. The contamination was extensive as over half of the exposed population became ill. A remarkable proportion of the population suffered also from joint symptoms within the following eight weeks [15]. Two questionnaire surveys done thirteen months apart, based on representative population samples, focused on short- and long-term health effects of the epidemic. The 15-month follow-up questionnaire study reported here describes the duration of gastrointestinal and arthritis-like symptoms after the epidemic.

53.6% of those having had gastroenteritis during the epidemic reported loose stools and abdominal pain after the acute disease and 42.7% loose stools and abdominal pain or distension. Although the prevalence decreased rapidly within five months, 10.9% was still having symptoms resembling IBS (loose stools and abdominal pain or distension) at 15 months.

Irritable bowel syndrome (IBS) is a condition characterized by altered bowel function and abdominal pain or discomfort. The studies on the prevalence of symptoms compatible with IBS in the general population have given widely variable figures depending on the definition used. For example, in a population-based Finnish study, the prevalence of IBS ranged from 5.1% to 16.2%, depending on diagnostic criteria [17]. According to a recent systematic review of worldwide IBS-studies, the pooled prevalence of IBS was 11.2% among adult subjects [18].

IBS occurring after an episode of infective gastroenteritis has been referred as post-infectious irritable bowel syndrome (PI-IBS). Generally about 10% of IBS-patients regard that their symptoms began after an infective gastroenteritis [3]. According to a meta-analysis of eight studies published until December 2005, the median prevalence of IBS was 9.8% in the infective gastroenteritis groups and 1.2% in the control groups [19]. The observed frequency of PI-IBS is likely to depend on the pathogen(s) involved. In Walkerton, Canada, the prevalence of IBS was 27.5% among adult subjects who had had gastroenteritis during the epidemic while the prevalence among those without gastroenteritis was 10.1% at 2–3 years after the outbreak [9]. Diarrhoea was found to be a more prominent feature in gastroenteritis-associated IBS than in the case of sporadic IBS. The prognosis of PI-IBS is thought to be better than IBS without infectious onset [3]; however, the condition can still take more than 8 years to resolve as was observed by the Walkerton group [3], [20]. If PI-IBS occurs after viral gastroenteritis, it is short lived and rarely lasts for 6 months or longer [4], [21].

Commonly used diagnostic criteria for IBS were not utilized in this study. However, abdominal pain or discomfort or distension and loose stools are the principal parts of these criteria. Analyzing these symptoms in such a combination makes it reasonably close to Manning 3 and/or Rome I criteria for IBS. The high prevalence of gastrointestinal symptoms within the first five months after the epidemic in our study may therefore indicate PI-IBS. However, at the end of the follow-up, the prevalence of these symptoms was 11.5% among adults ≥16 years of age. In the population-based study among adults in Finland, the prevalence of IBS was 9.7% according to the Manning 3 criteria [17]. Therefore, the remaining prevalence of gastrointestinal symptoms in the present study may be close to the natural occurrence of these symptoms.

Other reasons for long-lasting symptoms than PI-IBS must be considered in an epidemic where more than half of the exposed residents fell ill with gastroenteritis caused by mixed pathogens. There may have been prolonged circulation of the pathogens in the population leading to secondary infections. Bacterial stool cultures were extensively examined during the following months in the affected town. No excess frequency of positive findings could be noticed after the peak of the epidemic (Figure 2). This evidence does not support the prolonged circulation of bacterial pathogens in the population. Viral pathogens (especially, norovirus), are highly contagious and could well have started to spread horizontally after the outbreak. Such a possibility could not be excluded reliably because samples for viral diagnostics were not routinely taken during the study period. Giardiasis often causes long-lasting gastrointestinal symptoms. This epidemic was the first domestic *Giardia*-cluster in Finland [13]. The whole population was informed about the possibility of giardiasis and individuals were called for parasitological investigation if they suffered from prolonged symptoms. In spite of that, only 55 cases were found out of the 872 persons tested, a number that is probably too low to solely explain the magnitude of prolonged gastrointestinal symptoms.

As the data relies on self-reported symptoms, psychological factors like anger, anxiety or distress may have influenced the participants' expressions. The connection of psychological factors on the duration of IBS has been observed in epidemics caused by drinking water [20]. The public anger and anxiety in the town of Nokia was considerable for several months after the epidemic. Psychological influences of the incident on health experience will be investigated in another project and reported later.

Previously, we have reported a frequency of 6.7% of arthritis-like symptoms among the participants of the contaminated group within eight weeks from the incident [15]. According to the follow-up study, 31.8% of those who reported having had gastroenteritis during the epidemic announced subsequent arthritis-like symptoms. The explanation for the difference lies in the fact that joint symptoms in the follow-up study were asked for only from those with gastroenteritis and through a selection bias towards those with joint symptoms in the first study.

The arthritis-like symptoms were fairly longstanding. About a third of the symptoms were resolved within five months. Thereafter, the decline was slow and 19% of the respondents were still experiencing arthritis-like symptoms 15 months after the epidemic. In a previously published population-based study of verified ReA cases related to this outbreak, 21 cases of ReA were observed [14], mostly relatively mild cases. According to a follow-up study (one year) of this cohort, over a half of all the patients were still taking analgesics because of ReA symptoms and 33% was on antirheumatic medication [22]. This proportion of residual symptoms among verified ReA-cases is quite well in line with the findings of the present study.

ReA is a well-known complication of bacterial gastroenteritis [1]. *Giardia* has also been associated rarely with ReA [23]. To our knowledge, there are hardly any observations possibly linking rotavirus or norovirus to ReA. Considering this and the microbiological findings, we regard that campylobacter infections probably have been the major trigger of joint symptoms in this epidemic.

There are few population-based studies on the duration of self-reported joint symptoms following gastroenteritis epidemics. The previous literature deals with those fulfilling the criteria for ReA, either according to self-reporting or clinical judgement. In studies done in Finland, the average duration of ReA symptoms has been 3–5 months [1]. Symptoms persisting over six months have often been considered as a sign of chronic disorder. Development to the chronic condition has been reported in 12–16% of the cases [1]. The proportion of subjects with arthritis-like symptoms (19%), after 15 months in this study, is therefore, reasonably well in line with previous observations, especially, since this was a questionnaire study with no clinical verification of the symptoms.

The persistence of gastrointestinal symptoms correlated with the presence of arthritis-like symptoms (Table 2.). As PI-IBS and ReA are inflammatory disorders, pathogenesis of both gastrointestinal and arthritis-like symptoms may have common immunological mechanisms, although the genetic predisposition for these two conditions seems to be different [24].

There are some limitations of this study. First, although the initial sample for the first questionnaire was carefully randomized, there were three subsequent steps in which a selection bias could have taken place. They are responding to the first questionnaire, giving permission to be re-contacted, and finally responding to the follow-up survey (Table 1). The response rates of the first survey were good and the authors concluded that the survey was representative. For the follow-up survey, some selection in sample formation took place, but not any more in responding to the follow-up survey. Those with joint symptoms in the original cohort were more prone (OR 1.7, 95% CI 1.3–2.2) to participate in the 15-month follow-up. As the aim of the present study was to observe the duration of symptoms, selection towards participants who experienced symptoms during the outbreak does not seriously hamper the conclusions. Secondly, prolonged symptoms were asked only from those who fell ill with gastroenteritis during the outbreak. As one could assume, there were hardly any such cases in the control group and this made meaningful comparisons impossible. Thirdly, as the follow-up survey was done 15 months after the exposure, recalling the symptoms by those surveyed may have been difficult, thereby, creating a recall-bias of some degree. Fourthly, the outbreak and the faults causing it were widely discussed in the public media and several claims for compensation were made. Therefore, it is possible that some subjects with a disease that may have been potentially connected to the water contamination were prone to overweigh their symptoms.

The contamination of drinking water and the subsequent epidemic in the town of Nokia was a potentially dangerous situation. Large amounts of pathogens were distributed through household water and thousands of people fell ill. Given these circumstances, consequences could have been much more serious. In addition, more virulent bacterial strains, such as *Escherichia coli* O157:H7 were avoided. Despite the relatively favourable outcome of this epidemic, however, there were cases of people who suffered from consequences, especially, joint symptoms up to one year or more. In addition, individual patients have suffered from serious, prolonged, or permanent damages caused by joint destruction [22], IBS, or chronic fatigue syndrome.

In conclusion, prolonged gastrointestinal and joint symptoms were common among those who fell ill with gastroenteritis during the epidemic. The frequency of gastrointestinal symptoms declined during the follow-up period of 15 months. However, over half of the subjects with arthritis-like symptoms after the outbreak were still symptomatic at the end of the follow-up.

## References

[1] Leirisalo-Repo M (2005) Reactive arthritis. Scand J Rheumatol 34: 251–259. 16195157 10.1080/03009740500202540

[2] DuPont AW (2008) Postinfectious irritable bowel syndrome. Clin Infect Dis 46: 594–599. 18205536 10.1086/526774

[3] Spiller R, Garsed K (2009) Postinfectious irritable bowel syndrome. Gastroenterology 136: 1979–1988. 19457422 10.1053/j.gastro.2009.02.074

[4] Marshall JK, Thabane M, Borgaonkar MR, James C (2007) Postinfectious irritable bowel syndrome after a food-borne outbreak of acute gastroenteritis attributed to a viral pathogen. Clin Gastroenterol Hepatol 5: 457–460. 17289440 10.1016/j.cgh.2006.11.025

[5] Clark WF, Sontrop JM, Macnab JJ, Salvadori M, Moist L, et al (2010) Long term risk for hypertension, renal impairment, and cardiovascular disease after gastroenteritis from drinking water contaminated with Escherichia coli O157:H7: a prospective cohort study. Bmj 341: c6020. 21084368 10.1136/bmj.c6020 PMC3191723

[6] Garg AX, Moist L, Matsell D, Thiessen-Philbrook HR, Haynes RB, et al (2005) Risk of hypertension and reduced kidney function after acute gastroenteritis from bacteria-contaminated drinking water. Cmaj 173: 261–268. 15923490 10.1503/cmaj.050581 PMC1180655

[7] Garg AX, Pope JE, Thiessen-Philbrook H, Clark WF, Ouimet J (2008) Arthritis risk after acute bacterial gastroenteritis. Rheumatology (Oxford) 47: 200–204. 18184664 10.1093/rheumatology/kem339 PMC2876134

[8] Marshall JK (2009) Post-infectious irritable bowel syndrome following water contamination. Kidney Int Suppl: S42–43. 10.1038/ki.2008.618 19180133

[9] Marshall JK, Thabane M, Garg AX, Clark WF, Salvadori M, et al (2006) Incidence and epidemiology of irritable bowel syndrome after a large waterborne outbreak of bacterial dysentery. Gastroenterology 131: 445–450; quiz 660. 16890598 10.1053/j.gastro.2006.05.053

[10] Moist LM, Sontrop JM, Garg AX, Clark WF, Suri RS, et al (2009) Risk of pregnancy-related hypertension within five years of exposure to bacteria-contaminated drinking water. Kidney Int Suppl: S47–49. 10.1038/ki.2008.620 19180135

[11] Laine J, Huovinen E, Virtanen MJ, Snellman M, Lumio J, et al (2011) An extensive gastroenteritis outbreak after drinking-water contamination by sewage effluent, Finland. Epidemiol Infect 1–9. 10.1017/S0950268810002141 20843387

[12] Rasanen S, Lappalainen S, Kaikkonen S, Hamalainen M, Salminen M, et al (2010) Mixed viral infections causing acute gastroenteritis in children in a waterborne outbreak. Epidemiol Infect 138: 1227–1234. 20092670 10.1017/S0950268809991671

[13] Rimhanen-Finne R, Hanninen ML, Vuento R, Laine J, Jokiranta TS, et al (2010) Contaminated water caused the first outbreak of giardiasis in Finland, 2007: a descriptive study. Scand J Infect Dis 42: 613–619. 20429718 10.3109/00365541003774608

[14] Uotila T, Antonen J, Laine J, Kujansuu E, Haapala AM, et al (2011) Reactive arthritis in a population exposed to an extensive waterborne gastroenteritis outbreak after sewage contamination in Pirkanmaa, Finland. Scand J Rheumatol 40: 358–362. 21679096 10.3109/03009742.2011.562533

[15] Laine J, Uotila T, Antonen J, Korpela M, Kujansuu E, et al (2012) Joint symptoms after a large waterborne gastroenteritis outbreak–a controlled, population-based questionnaire study. Rheumatology (Oxford) 51: 513–518. 22120464 10.1093/rheumatology/ker320

[16] Maunula L, Kllemola P, Kauppinen A, Söderberg K, Nguyen T, et al (2009) Enteritic viruses in a large waterborne outbreak of acute gastroenteritis in Finland. Food Environ Virol 1: 31–36.

[17] Hillila MT, Farkkila MA (2004) Prevalence of irritable bowel syndrome according to different diagnostic criteria in a non-selected adult population. Aliment Pharmacol Ther 20: 339–345. 15274671 10.1111/j.1365-2036.2004.02034.x

[18] Lovell RM, Ford AC (2012) Global Prevalence of, and Risk Factors for, Irritable Bowel Syndrome: a Meta-analysis. Clin Gastroenterol Hepatol 10.1016/j.cgh.2012.02.029 22426087

[19] Halvorson HA, Schlett CD, Riddle MS (2006) Postinfectious irritable bowel syndrome–a meta-analysis. Am J Gastroenterol 101: 1894–1899; quiz 1942. 16928253 10.1111/j.1572-0241.2006.00654.x

[20] Marshall JK, Thabane M, Garg AX, Clark WF, Moayyedi P, et al (2010) Eight year prognosis of postinfectious irritable bowel syndrome following waterborne bacterial dysentery. Gut 59: 605–611. 20427395 10.1136/gut.2009.202234

[21] Porter CK, Faix DJ, Shiau D, Espiritu J, Espinosa BJ, et al (2012) Postinfectious gastrointestinal disorders following norovirus outbreaks. Clin Infect Dis 55: 915–922. 22715178 10.1093/cid/cis576

[22] Uotila TM, Antonen JA, Paakkala AS, Mustonen JT, Korpela MM (2013) Outcome of reactive arthritis after an extensive Finnish waterborne gastroenteritis outbreak: a 1-year prospective follow-up study. Clin Rheumatol 10.1007/s10067-013-2247-x 23559390

[23] Morris D, Inman RD (2012) Reactive arthritis: developments and challenges in diagnosis and treatment. Curr Rheumatol Rep 14: 390–394. 22821199 10.1007/s11926-012-0280-4

[24] Nielsen H, Steffensen R, Ejlertsen T (2012) Risk and prognosis of campylobacteriosis in relation to polymorphisms of host inflammatory cytokine genes. Scand J Immunol 75: 449–454. 22229864 10.1111/j.1365-3083.2012.02678.x

